# CT differentiation of the oncocytoma and renal cell carcinoma based on peripheral tumor parenchyma and central hypodense area characterisation

**DOI:** 10.1186/s12880-023-00972-0

**Published:** 2023-01-27

**Authors:** Jianyi Qu, Qianqian Zhang, Xinhong Song, Hong Jiang, Heng Ma, Wenhua Li, Xiaofei Wang

**Affiliations:** 1 Yuhuangding Hospital, Qingdao University School of Medicine, Shandong Yantai, China; 2 Xinhua Hospital, Shanghai Jiaotong University School of Medicine, Shanghai, China; 3 Yantaishan Hospital, Binzhou Medical University, Shandong Yantai, China

**Keywords:** Oncocytoma, Renal cell carcinoma, Central hypodense area, Enhancement inversion, Peripheral tumor parenchyma, CT

## Abstract

**Background:**

Although the central scar is an essential imaging characteristic of renal oncocytoma (RO), its utility in distinguishing RO from renal cell carcinoma (RCC) has not been well explored. The study aimed to evaluate whether the combination of CT characteristics of the peripheral tumor parenchyma (PTP) and central hypodense area (CHA) can differentiate typical RO with CHA from RCC.

**Methods:**

A total of 132 tumors on the initial dataset were retrospectively evaluated using four-phase CT. The excretory phases were performed more than 20 min after the contrast injection. In corticomedullary phase (CMP) images, all tumors had CHAs. These tumors were categorized into RO (*n* = 23), clear cell RCC (ccRCC) (*n* = 85), and non-ccRCC (*n* = 24) groups. The differences in these qualitative and quantitative CT features of CHA and PTP between ROs and ccRCCs/non-ccRCCs were statistically examined. Logistic regression filters the main factors for separating ROs from ccRCCs/non-ccRCCs. The prediction models omitting and incorporating CHA features were constructed and evaluated, respectively. The effectiveness of the prediction models including CHA characteristics was then confirmed through a validation dataset (8 ROs, 35 ccRCCs, and 10 non-ccRCCs).

**Results:**

The findings indicate that for differentiating ROs from ccRCCs and non-ccRCCs, prediction models with CHA characteristics surpassed models without CHA, with the corresponding areas under the curve (AUC) being 0.962 and 0.914 versus 0.952 and 0.839 respectively. In the prediction models that included CHA parameters, the relative enhancement ratio (RER) in CMP and enhancement inversion, as well as RER in nephrographic phase and enhancement inversion were the primary drivers for differentiating ROs from ccRCCs and non-ccRCCs, respectively. The prediction models with CHA characteristics had the comparable diagnostic ability on the validation dataset, with respective AUC values of 0.936 and 0.938 for differentiating ROs from ccRCCs and non-ccRCCs.

**Conclusion:**

The prediction models with CHA characteristics can help better differentiate typical ROs from RCCs. When a mass with CHA is discovered, particularly if RO is suspected, EP images with longer delay scanning periods should be acquired to evaluate the enhancement inversion characteristics of CHA.

## Background

Renal oncocytoma (RO) is a benign solid renal tumor that account for 3–7% of all renal tumors, while renal cell carcinoma (RCC) is the most common malignant renal tumor [1]. The optimum therapy and surgical prognosis of RO and RCC vary significantly because of their different biological features. As a benign tumor, RO is routinely followed by active monitoring or treated with partial nephrectomy [2]. Thus, the ability to properly differentiate between ROs and RCCs is critical.

A variety of quantitative and qualitative contrast-enhanced CT features, such as enhancement degree, enhancement pattern, tumor heterogeneity, cystic components, and central scar, have been used in several studies to distinguish ROs from RCCs and have shown some value in differential diagnosis [3–6]. Meanwhile, these studies also show that ROs and RCCs still overlap in CT features. Both clear cell RCCs (ccRCCs) and ROs show strong enhancement in the corticomedullary phase (CMP) images due to a high capillary network [5]. Because of comparable origin and often solid growth patterns, ROs and chromophobe RCCs (chRCCs) also exhibit similar imaging features in many respects, such as rare cystic components and common central scars [7, 8]. Papillary RCCs (pRCCs) are relatively easy to distinguish from ROs because of their weak and progressive enhancement on contrast-enhanced CT [5]. In addition, given that pRCCs and chRCCs were both hypovascular tumors and had a better prognosis than ccRCCs, they were often classified as a non-ccRCC group in many studies [5, 9]. In summary, differentiating ROs from RCCs remains challenging, and many patients with ROs undergo unnecessary radical nephrectomy.

Typical ROs have previously been identified on CT scans to have a central hypodense area (CHA), also known as a central scar[5]. However, it is not sufficiently specific since the central scar can also be seen in a small proportion of RCCs, and the central necrosis occurring inside RCCs may mimic a central scar [7–10]. An earlier investigation has shown that the assessment of the enhancement inversion of CHA in excretory phase (EP) images was valuable for distinguishing ROs with a central scar from ccRCCs [10], suggesting the importance of further analysis of the CHA imaging features.

The aim of the research was thus to further retrospectively investigate the performance of four-phase CT in differentiating typical ROs with CHA from RCCs based on both qualitative and quantitative CT features of CHA and peripheral tumor parenchyma (PTP).

## Methods

### Patient cohort

Our institution’s radiography and pathology databases were searched between June 2013 and January 2021 to identify all RO, ccRCC, chRCC, and pRCC cases. Two radiologists with four and six years of professional experience reviewed these patients. The identified cases were evaluated according to the inclusion criteria listed below: (a) All patients received a four-phase CT scan that included a preoperative CMP, nephrographic phase (NP), EP, and pre-contrast phase. The EP images were collected more than 20 min after the contrast injection. (b) All of the cases were histologically identified after partial or radical nephrectomy. (c) All patients were evaluated to ensure that only tumors visibly displaying stellate or irregular CHA in CMP imaging were included in the study. Eventually, the final initial dataset included 132 tumors from 132 patients, of which 23 were ROs, 85 were ccRCCs, 18 were chRCCs and 6 were pRCCs. Ultimately, the study population comprised 70 men and 62 women; the mean age ± standard deviations (SDs) were 58.1 ± 10.5 years. The participants were separated into three groups: RO (*n* = 23), ccRCC (*n* = 85), and non-ccRCC (*n* = 24).

Subsequently, we included a validation dataset to validate the final prediction models that included CHA features. All of the validation dataset’s samples were collected from a single institution throughout the same time frame and using the same inclusion criteria as the original dataset. The validation dataset comprised 28 men and 25 women; the mean age ± SDs were 57.1 ± 10.1 years. They were also separated into three groups: RO (*n* = 8), ccRCC (*n* = 35), and non-ccRCC (*n* = 10). Among them, the non-ccRCC group included 8 chRCCs and 2 pRCCs.

### CT protocol

The 64 or 256 detector row helical scanners (Philips Brilliance) were used to perform all CT examinations. Patients were told to hold their breath while having a CT scan. The parameters were: 150–250 mA tube current, 120 kV tube voltage, 5 mm section thickness, and 5 mm reconstruction interval. High-pressure automated injectors were used to deliver 80 to 100ml of iohexol (General Electric Pharmaceuticals Shanghai Co., Ltd.) into the antecubital vein. The injection rate was 5ml/s. The renal CMP, NP and EP images were obtained at 25–30 s, 60–90 s and more than 20 min after contrast injection, respectively.

### CT features analysis

On the picture archiving and communication system workstation, two additional blinded radiologists (9 and 20 years of experience in abdominal imaging, respectively) analyzed and measured all identified cases on the initial dataset. All qualitative and quantitative CT features were assessed and quantified throughout the axial images.

Localization of the tumor (left kidney or right kidney), the growth pattern of the tumor (endophytic, meaning > 50% within renal parenchyma, or exophytic, meaning > 50% outside renal parenchyma), qualitative CT features of CHA (enhancement inversion, calcification, and typical stellate pattern), and qualitative CT features of PTP (persistent low sign, pseudocapsule sign, calcification) were all included in the qualitative CT features. When CHA enhanced slowly in a centripetal way after contrast injection and exhibited higher attenuation than PTP in EP, an enhancement inversion was considered present. The term “complete enhancement inversion” referred to when the entire CHA was enhanced and showed higher attenuation than PTP in EP. The term “incomplete enhancement inversion” was used to refer to the fact that in EP, the CHA was only peripherally enhanced and exhibited higher attenuation than PTP [10]. According to Giambelluca et al. [11], the presence of the typical stellate pattern of CHA was subjectively assessed in CMP or NP. Calcification within CHA or PTP was recorded independently. A high or low attenuation rim encircling the tumor was classified as a pseudocapsule sign. A persistent low sign was characterized as a localized hypodensity at the same PTP position in all contrast-enhanced phases [12]. Two radiologists independently examined these qualitative CT findings, and the statistical analysis was based on the consensus of two readers.

Radiologists measured the biggest diameter of CHA twice in NP images and recorded the average value as the CHA’s long-axis diameter. The biggest diameter of CHA was divided by the largest diameter perpendicular to it to arrive at the long-to-short-axis ratio (LSR). It was computed by comparing the biggest diameters of CHA to the largest diameters of tumors to calculate the long-tumor-axis ratio (LTR). The two radiologists then agreed on the tumor location that showed the greatest enhancement during the three contrast-enhanced phases. The attenuation value of tumors (AVT) was determined by placing 8-15mm^2^ elliptical or circular regions of interest (ROI) in these locations. In addition, the ROI was established in the nearby renal cortex to estimate the attenuation value of the cortex (AVC). The ROI’s position remained constant throughout all scan phases. Each parameter was measured twice using a cursor of the same design and size, and the average value was calculated from the two measurements. According to the following formula, the relative enhancement ratio (RER) of the tumor was calculated:

(AVT/AVC) ×100%.

An independent radiologist with 4 years of experience performed the same analyses and measurements on the validation dataset.

The gold standard was determined based on pathologic results.

### Statistical analysis

The SPSS for Windows statistical analysis program was used for the evaluation (ver. 25.0; IBM Inc). Chi-square test or Fisher exact test were used to compare the qualitative data. The Kolmogorov-Smirnov test was used to determine the normality of quantitative data, including the largest diameter of tumor, the CHA’s long-axis diameter, LSR, LTR, AVT, and RER. The independent sample *t*-test and Mann-Whitney *U* test were used for quantitative data that followed normal distribution and those that did not, respectively. A *p*-value of < 0.05 was considered statistically significant. A logistic regression analysis was then performed using the parameters with *p* < 0.05. First, to distinguish RO from the two RCC groups, the parameters with *p* < 0.05 other than CHA features (enhancement inversion, calcification within CHA, typical stellate pattern, the CHA’s long-axis diameter, LSR and LTR) were screened, and corresponding prediction models were developed. Then, using CHA characteristics with *p* < 0.05, we developed prediction models and screened more key elements that distinguished RO from two RCC groups. Each model’s predictive ability was assessed through its positive predictive value (PPV), negative predictive value (NPV), sensitivity, specificity, and accuracy, as well as the area under the curve (AUC). The above predictive models, which used CHA characteristics developed from the initial dataset, were tested on the validation dataset. The validation cohort’s sensitivity, specificity, PPV, NPV, accuracy, and AUC were then determined.

## Results

### Qualitative analysis

**Fig. 1:**
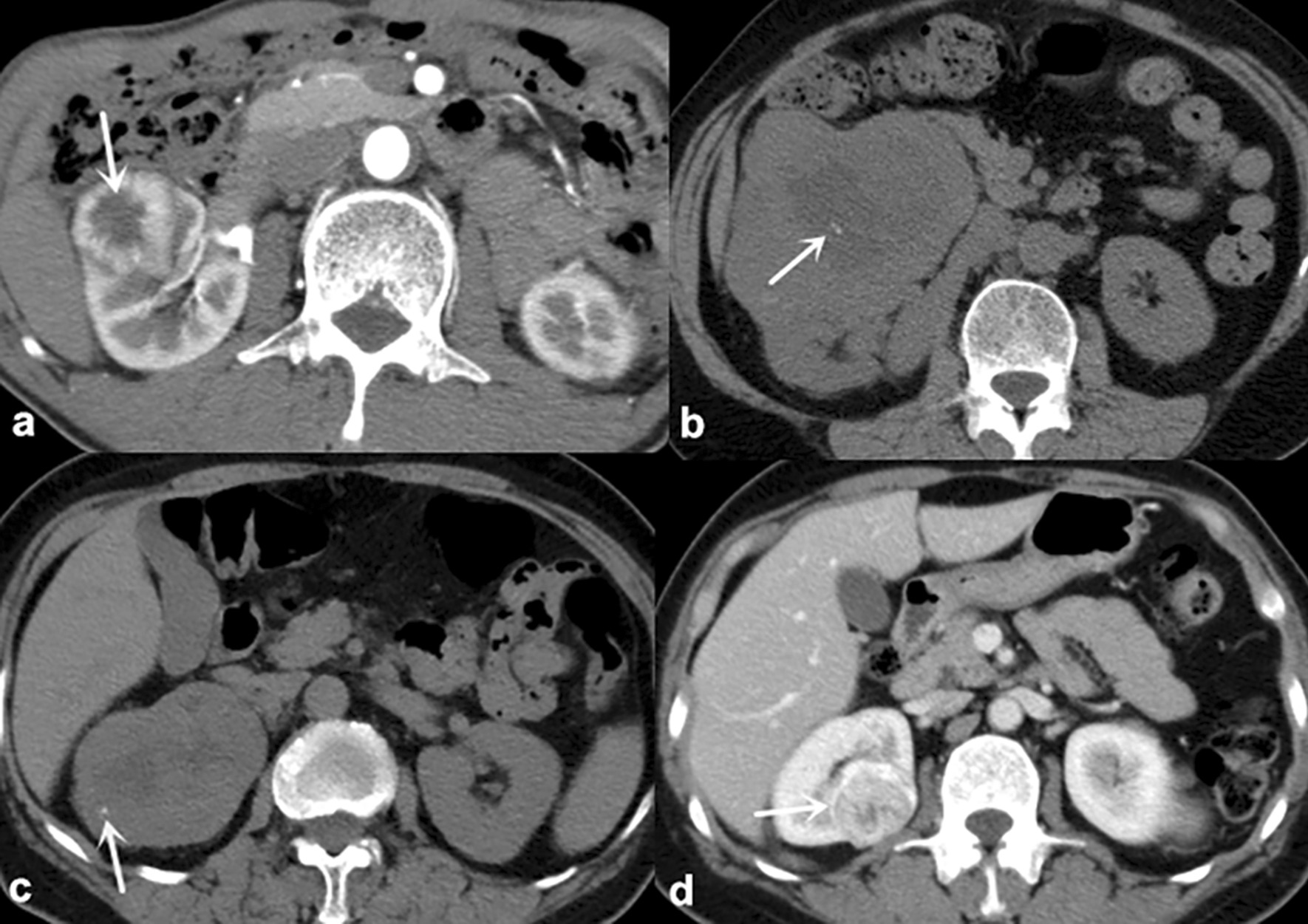
Schematic diagram of the qualitative features. **a** 66-year-old male with RO. The CHA of tumor shows as a typical stellate pattern (arrow). **b** A 59-year-old female with RO. Calcification within CHA is observed(arrow). **c** A 62-year-old female with ccRCC. Calcification within PTP is observed(arrow). **d** A 55-year-old female with ccRCC. Pseudocapsule sign is discovered at the edge of tumor (arrow). * CHA* Central hypodense area, *ccRCC* Clear cell renal cell carcinoma,  *LSR* Long-to-short-axis ratio, *PTP* Peripheral tumor parenchyma, *RO* Renal oncocytoma

**Fig. 2:**
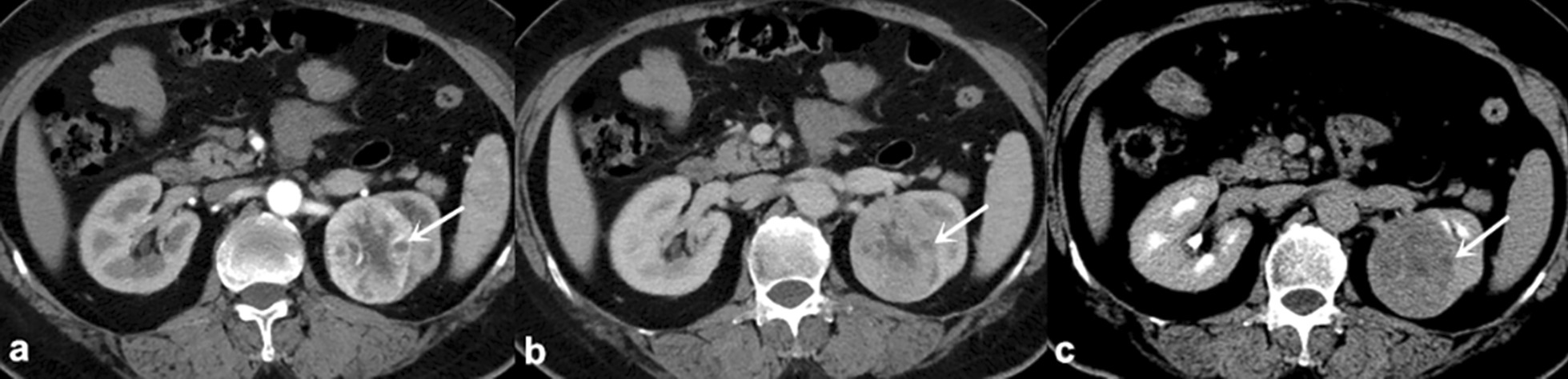
Persistent low sign of ccRCC in a 60-year-old female . ** a**–**c** The CMP, NP and EP images show a focal hypodensity at the same location of PTP (arrows), except for CHA. * CMP* Corticomedullary phase,  *ccRCC* Clear cell renal cell carcinoma,  *CHA* Central hypodense area,  *EP* Excretory phase,  *NP* Nephrographic phase,  *PTP* Peripheral tumor parenchyma

**Fig. 3:**
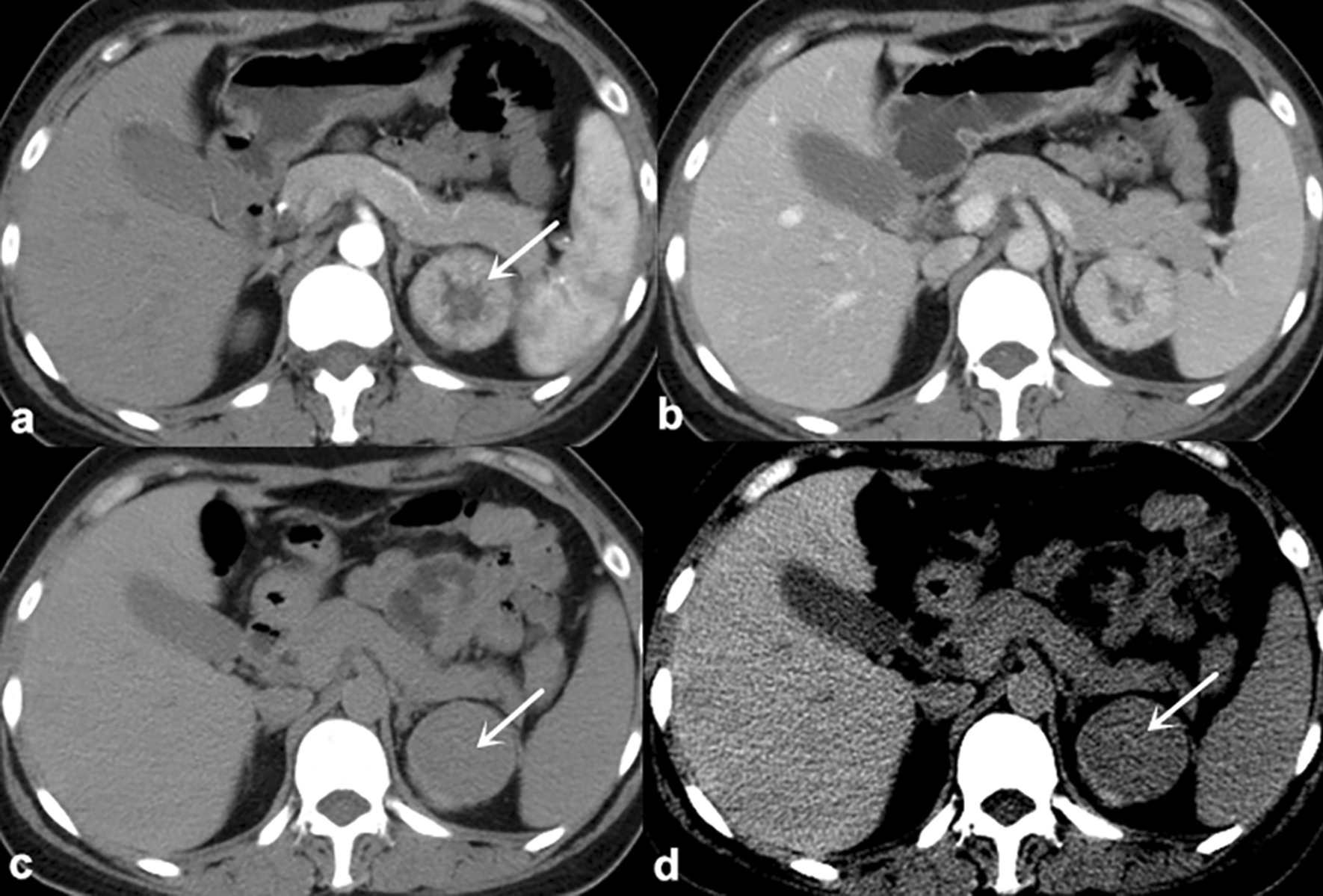
Complete enhancement inversion of RO in a 48-year-old female. **a** The CMP image show a 4.1-cm-diameter mass with CHA(arrow). **b** and **c** The NP and EP images show that the CHA enhance slowly in a centripetal manner. The EP image shows that enhancement inversion is complete(arrow). **d** The EP image with different windowing can better display the enhancement inversion of the CHA(arrow). * CMP* Corticomedullary phase,  *CHA* Central hypodense area,  *EP* Excretory phase,  *NP* Nephrographic phase,  *RO* Renal oncocytoma

**Fig. 4:**
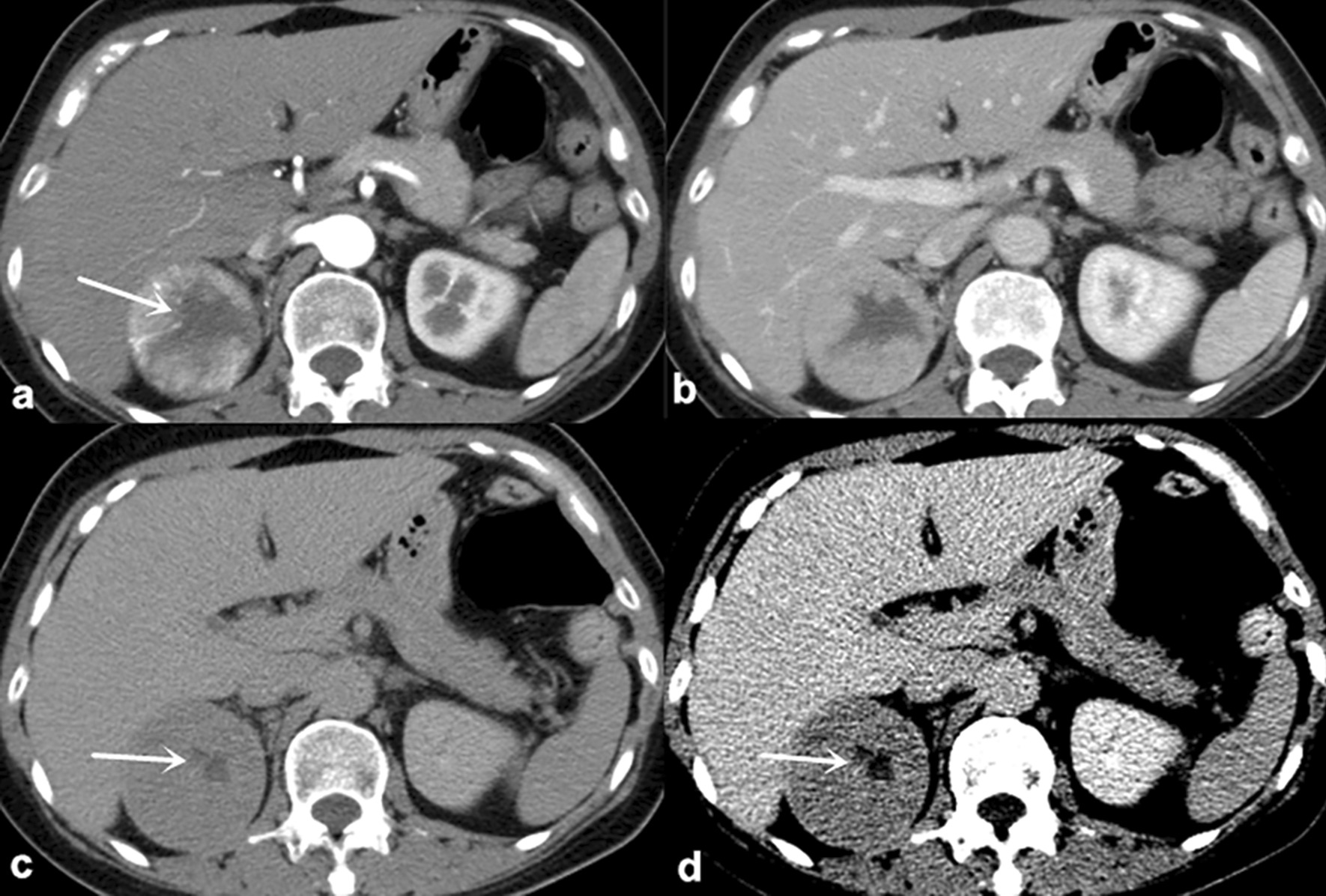
Incomplete enhancement inversion of chRCC in 56-year-old female. **a** The CMP image show a 5.8-cm-diameter mass with CHA(arrow). **b** and **c** The NP and EP images show that the CHA appears progressive enhancement in a centripetal manner except in inner portion. The enhancement inversion is incomplete. Note the higher enhancement at junction between CHA and PTP(arrow). **d** The EP image with different windowing can better display the incomplete enhancement inversion of the CHA(arrow). * CMP* Corticomedullary phase,  *ccRCC* Clear cell renal cell carcinoma,  *CHA* Central hypodense area,  *chRCC* Chromophobe renal cell carcinoma,  *EP* Excretory phase,  *NP* Nephrographic phase,  *PTP* Peripheral tumor parenchyma

The results of qualitative data are shown in Table 1; Figs. 1, 2, 3 and 4. RO and ccRCC had significant differences in enhancement inversion and persistent low sign (*p* < 0.05). RO and non-ccRCC revealed significant differences in the typical stellate pattern, enhancement inversion and calcification(PTP) (*p* < 0.05). The remaining parameters between RO and RCC overlapped significantly.

**Table 1:** The comparative analysis on qualitative features of ROs and RCCs

Variables	ROs (*n* = 23)	CcRCCs (*n* = 85)	*P* value	Non-ccRCC (*n* = 24)	*P* value
Location					
Left kidney Right kidney	10(43.48) 13(56.52)	45(52.94) 40(47.06)	0.421	15(62.50) 9(37.50)	0.191
Growth pattern					
Endophytic Exophytic	10(43.48) 13(56.52)	27(31.76) 58(68.24)	0.294	10(41.67) 14(58.33)	0.900
Typical stellate pattern					
Present Absent	10(43.48) 13(56.52)	21(24.71) 64(75.29)	0.077	4(16.67) 20(83.33)	0.045
Enhancement inversion					
Complete Incomplete None	15(65.22) 8(34.78) 0	21(24.71) 51(60.00) 13(15.29)	0.001	4(16.67) 15(62.50) 5(20.83)	0.001
Calcification(CHA)					
Present Absent	3(13.04) 20(86.96)	4(4.71) 81(95.29)	0.150	2(8.33) 22(91.67)	0.601
Persistent low sign					
Present Absent	2(8.70) 21(91.30)	32(37.65) 53(62.35)	0.008	7(29.17) 17(70.83)	0.075
Calcification(PTP)					
Present Absent	0 23(100.00)	6(7.06) 79(92.94)	0.190	7(29.17) 17(70.83)	0.005
Pseudocapsule sign					
Present Absent	7(30.43) 16(69.57)	35(41.18) 50(58.82)	0.349	8(33.33) 16(66.67)	0.831

*ccRCC* Clear cell renal cell carcinoma,  *CHA* Central hypodense area,  *PTP* Peripheral tumor parenchyma,  *RCC* Renal cell carcinoma,  *RO* Renal oncocytoma

Data in parentheses are percentages

### Quantitative analysis

The quantitative analysis results are shown in Table 2. RO and ccRCC had significant differences in the CHA’s long-axis diameter, LSR, LTR, AVT in CMP, and RER in CMP (p < 0.05). In all contrast-enhanced phases, RO and non-ccRCC revealed significant differences in AVT and RER (*p* < 0.05). The remaining parameters between RO and RCC overlapped significantly.

**Table 2:** The comparative analysis on qualitative features of ROs and RCCs

Variables	ROs (*n* = 23)	CcRCCs (*n* = 85)	*P* value	Non-ccRCCs (*n* = 24)	*P* value
The largest diameter of tumor (cm)	4.47 ± 2.11	4.74 ± 1.50	0.156	5.40 ± 1.94	0.079
The CHA’s long-axis diameter(cm)	2.43 ± 1.32	3.05 ± 1.29	0.046	3.12 ± 2.12	0.317
LSR	1.43 ± 0.36	1.58 ± 0.39	0.048	1.66 ± 0.50	0.062
LTR	0.54 ± 0.18	0.63 ± 0.14	0.013	0.54 ± 0.21	0.686
AVT					
PCP	38.35 ± 4.74	38.86 ± 6.25	0.717	41.08 ± 8.36	0.148
CMP	139.17 ± 24.97	189.20 ± 44.46	< 0.001	119.04 ± 50.14	0.015
NP	125.74 ± 21.86	121.73 ± 22.21	0.443	95.29 ± 26.38	< 0.001
EP	62.87 ± 10.64	60.56 ± 9.69	0.324	58.46 ± 10.16	0.031
RER					
PCP	1.09 ± 0.17	1.15 ± 0.22	0.372	1.22 ± 0.23	0.418
CMP	0.86 ± 0.12	1.18 ± 0.23	< 0.001	0.67 ± 0.27	0.004
NP	0.77 ± 0.13	0.75 ± 0.12	0.589	0.57 ± 0.15	< 0.001
EP	0.74 ± 0.11	0.73 ± 0.17	0.688	0.67 ± 0.14	0.008

*AVT* Attenuation value of tumor,  *ccRCC* Clear cell renal cell carcinoma,  *CHA* Central hypodense area,  *CMP* Corticomedullary phase,  *EP* Excretory phase,  *LSR* Long-to-short-axis ratio. *LTR* Long-tumor-axis ratio,  *NP* Nephrographic phase,  *PCP* Pre-contrast phase,  *RO* Renal oncocytoma,  *RCC* Renal cell carcinoma,  *RER* Relative enhancement ratio

All numeric variables are expressed as means ± standard deviation

### Multivariate analysis and prediction models

**Fig. 5:**
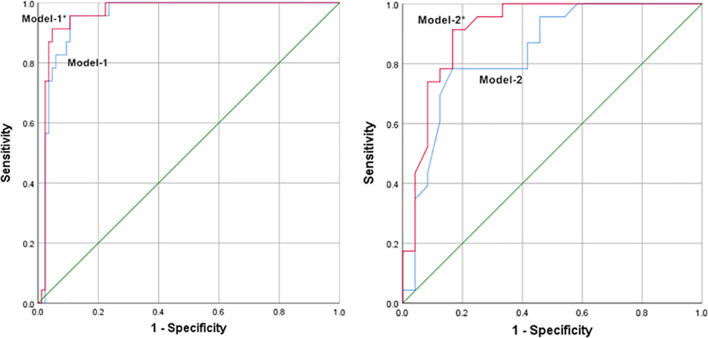
Receiver operating characteristic curves of the prediction models. Model-1 and Model-2 are predictive models excluding CT features of CHA for differentiating RO from ccRCC and non-ccRCC, respectively. Model-1* and Model-2* are predictive models including CT features of CHA for differentiating RO from ccRCC and non-ccRCC, respectively. * ccRCC* Clear cell renal cell carcinoma,  *CHA* Central hypodense area,  *RO* Renal oncocytoma,  *RCC* Renal cell carcinoma

The results of the logistic regression analysis are shown in Table 3. The table showed that one of the most important characteristics of PTP for differentiation were the enhancement of ccRCC in CMP and non-ccRCC in NP. The most important CHA component for differentiation was enhancement inversion. Table 4 summarizes the prediction models that distinguished RO and RCC by excluding or including CHA characteristics and the test results for the prediction models that did so on the validation dataset. Prediction models that used CHA features outperformed the models without CHA (Fig. 5). Meanwhile, the prediction models including CHA features exhibited similar diagnostic performance on the validation dataset.

**Table 3:** Multivariate regression analysis excluding or including CHA features for differentiation of ROs and RCCs

Model	Coefficient	Odds ratio	95% CI (odds ratio)	*P* value
Differentiation of ROs and ccRCCs excluding CT features of CHA				
Constant Persistent low sign AVT in CMP RER in CMP	− 8.052 − 1.893 0.025 6.616	0.151 1.026 747.002	0.026–0.880 1.001–1.051 10.770-51814.956	0.036 0.042 0.002
Differentiation of ROs and ccRCCs including CT features of CHA				
Constant Enhancement inversion RER in CMP	− 9.218 − 2.106 0.023	0.122 706.727	0.018–0.820 7.457-66979.129	0.030 0.005
Differentiation of ROs and non-ccRCCs excluding CT features of CHA				
Constant RER in NP	6.539 − 9.737	0.000	0.000-0.014	< 0.001
Differentiation of ROs and non-ccRCCs including CT features of CHA				
Constant Enhancement inversion RER in NP	6.339 − 1.690 −8.484	0.184 0.000	0.038−0.900 0.000−0.067	0.037 0.004

*AVT* Attenuation value of tumor. *ccRCC* Clear cell renal cell carcinoma, *CHA* Central hypodense area, *CMP* Corticomedullary phase, *LSR* Long-to-short-axis ratio, *NP* Nephrographic phase, *RO* Renal oncocytoma, *RCC* Renal cell carcinoma, . RER: relative enhancement ratio

**Table 4:** Diagnostic performance of predictive models for differentiation of ROs and RCCs

Model parameter	Model−1	Model−1^*^	Model−1^*^_validation_	Model−2	Model−2^*^	Model−2^*^_validation_
Sensitivity	0.783(18/23)	0.826(19/23)	0.875(7/8)	0.783(18/23)	0.826(19/23)	0.750(6/8)
Specificity	0.953(81/85)	0.965(82/85)	0.829(29/35)	0.833(20/24)	0.875(21/24)	0.900(9/10)
PPV	0.818(18/22)	0.864(19/22)	0.538(7/13)	0.818(18/22)	0.864(19/22)	0.857(6/7)
NPV	0.942(81/86)	0.953(82/86)	0.967(29/30)	0.800(20/25)	0.840(21/25)	0.818(9/11)
Accuracy	0.917(99/108)	0.935(101/108)	0.837(36/43)	0.809(38/47)	0.851(40/47)	0.833(40/47)
AUC	0.952	0.962	0.936	0.839	0.914	0.938
AUC(95% CI)	0.912–0.992	0.925–0.999	0.860-1.000	0.722–0.955	0.828-1.000	0.825-1.000

Model-1 and Model-2 are predictive models excluding CT features of CHA for differentiating RO from ccRCC and non-ccRCC, respectively. Model-1* and Model-2* are predictive models including CT features of CHA for differentiating RO from ccRCC and non-ccRCC, respectively

Model−1^*^_validation_ and Model−2^*^_validation_ are the test results of Model-1^*^ and Model-2^*^ on the validation dataset, respectively

Values are ratios of the numerator and denominator in parentheses

*AUC* Area under curve,  *CI* Confidence interval,  *CHA* Central hypodense area,  *ccRCC* Clear cell renal cell carcinoma,  *NPV* Negative predictive value, *PPV* Positive predictive value,  *RO* Renal oncocytoma,  *RCC* Renal cell carcinoma

## Discussion

The current research found that logistic regression models built using qualitative and quantitative CT features of PTP can distinguish RO from ccRCC and non-ccRCC with moderate to excellent accuracy. Adding CT characteristics of CHA may boost diagnostic performance even further, particularly when distinguishing RO from non-ccRCC.

Although the central scar is an essential imaging feature of ROs, its CT characteristics have not been well investigated. Our research yielded some significant findings. Firstly, although there was no statistical significance between ROs and ccRCCs, the typical stellate pattern was more prevalent in ROs (10/23, 43.48%) than in ccRCCs (21/85, 24.71%, *p* = 0.077) and non-ccRCCs (4/24, 16.67%, *p* = 0.045), which may explain why the central stellate scar was first recognized as a distinct indication of ROs [13]. Secondly, ROs had a lower LSR than ccRCCs. A large LSR indicates a broader CHA width and length disparity, indicating a more elongated and eccentric shape [14, 15]. To our knowledge, this parameter has not been used in previous studies regarding central scars. Additionally, LTR and the CHA’s long-axis diameter of ROs were both smaller than those of ccRCCs. It is significant to remember that due to inter- and intraobserver variation, all of these features may be subjective. Additionally, despite being clinically significant (all *p* < 0.05), the difference between the groups was not very obvious. Thankfully, they were left out of the final prediction models that distinguished between ROs and ccRCCs, indicating that such parameters are not strictly necessary for routine measurement in our clinical practice. Thirdly, unlike the previous study that included only RO and ccRCC cases [10], the current study showed that a longer delay scanning period (> 20 min after contrast injection) was valuable not only for distinguishing ROs from ccRCCs, but also ROs from non-ccRCCs. When scanning was delayed longer, the RO group was more likely to have a complete enhancement inversion than the two RCC groups. In addition, all ROs showed complete or incomplete enhancement inversion in EP, indicating that the lack of enhancement inversion was a negative predictor of ROs.

Our study showed that calcification and persistent low sign within PTP were important negative predictors of ROs. Calcification within PTP was found in 11.92% (13/109) of all RCCs, but this was not seen in any RO. It has been reported that calcification in RO is relatively rare and typically present within the central scar, which is consistent with our study [1]. Although calcification was not included in our logistic regression models, the possibility of RO was close to zero when calcification is discovered in PTP. The persistent low sign was found in only 8.70% (2/23) of ROs but in 35.78% (39/109) of all RCCs (*p* = 0.011), which was consistent with RO presenting as a homogeneous mass without necrosis or cystic degeneration [16]. Additionally, the current analysis did not examine the imaging characteristic known as “segmental enhancement inversion,“ which was first observed in homogenous renal masses less than 4 cm in diameter without a central scar in early EP (delayed 2 to 3 min) [17]. In comparison, the current investigation did not limit the homogeneity or size of the lesions and employed a longer delay scanning duration.

Several studies have examined various quantitative measures and correction strategies to distinguish ROs from RCCs on multiphase CT [3, 4, 18–20]. In the current study, AVT and RER in CMP of RO were significantly lower than those of ccRCC, comparable to previous studies [10, 21]. On the other hand, chRCC and RO have comparable imaging characteristics and a shared cellular origin [11]. An early study revealed that 30–40% of chRCCs also have central scars [22]. As a result, the differential diagnosis of RO and chRCC using non-invasive imaging approaches has long been a research focus. However, because of the small number of patients (we only examined pRCCs and chRCCs with CHA) and the fact that both pRCCs and chRCCs were hypovascular tumors with a better prognosis than ccRCCs, we designated them as non-ccRCCs in the present study. According to our findings, in all contrast-enhanced phases, AVT and RER demonstrated significant differences between RO and non-ccRCC, notably in NP. The result was compatible with imaging aspects of mild and moderate enhancement of pRCC and chRCC reported in previous studies [8, 23–26].

Although the present study showed that several CT features of PTP and CHA were confirmed to be associated with RO, none of the findings was sufficiently accurate. Therefore, we combined several features to construct logistic regression models for improving diagnostic accuracy. To more accurately identify typical RO with CHA from ccRCC and non-ccRCC, we innovatively included CT features of CHA and constructed prediction models including and excluding CHA features, respectively. Our study showed that the diagnostic accuracy in differentiating RO and the two RCC groups was further improved when CHA features were included. According to the findings, enhancement inversion was the only CHA element in prediction models that included CHA features, while other CHA features were all excluded. This indicated the importance of employing a longer delay scanning time to assess the enhancement inversion of CHA. However, 20 min is longer than the 7–10 min delay of usual kidney CT protocols[27]. A 20 min delayed phase can negatively affect the throughput of the CT room, even if the patient leaves the CT room and comes back after a certain time. So the applicability and versatility of this discovery can be affected by the long-delayed protocol. Therefore, in daily practice, we suggest that the longer delay scanning period may be further performed only when a mass with CHA is found, especially if RO is suspected. Moreover, Cornelis et al.[28] looked into the enhancement features of central high T2-weighted signal of ROs and RCCs on the late enhanced MRI (delayed > 5 min), indicating that the enhancement features in the central area helped distinguish ROs from RCCs. Therefore, we think it’s important to conduct additional research to assess CHA features using CT scans with a shorter delay of 7–10 min. In addition, since we only dealt with ROs and RCCs with CHA, the sample of the study might not reflect the prevalence of the actual disease. If samples were obtained in the same manner from different institutions in the same period, the RO and RCC ratios may vary, and such different prevalence rates may exhibit different diagnostic capabilities when the same model is applied. Therefore, we included a validation dataset to validate the prediction models that included CHA features. According to the findings, the models exhibited similar diagnostic performance on the validation dataset. Compared with previous studies’ prediction models, our models innovatively contained CHA features and showed a better classification efficiency[3, 4, 6]. Even though numerous studies have demonstrated the value of radiomics in distinguishing benign from malignant renal tumors[27, 29, 30], such as the study by Li et al.[27] demonstrating that a CT-based radiomics nomogram had similar efficiency to our model in classifying ROs and ccRCCs, texture analysis has not yet been widely implemented in daily routine diagnosis. In general, our work yielded notable results deserving further investigation, which can serve as one of the essential diagnostic criteria for the classification of ROs and RCCs by several imaging modalities, such as multi-parametric MRI and ^99m^Tc-Sestamibi SPECT/CT[28, 31, 32].

Our research has some drawbacks. First, the study is prone to selection bias due to its retrospective methodology. Second, we only looked at CHA-positive tumors and excluded ROs and RCCs that had a uniform appearance, which reduced the number of ROs and non-ccRCCs to a very modest number. In addition, removing RCCs that were not linked with a characteristic central necrosis or scar resulted in an increased RO ratio. Third, some qualitative and quantitative elements, such as enhancement inversion, LSR, and LTR, might be subjective due to the inter-observer variation. Fourth, because of the different treatment methods but similar imaging characteristics, previous researches on the differentiation between malignant and benign renal masses mostly focused on renal masses with a diameter of 4 cm or smaller. However, limiting the size of the lesions will reduce the sample size to a great extent, preventing effective imaging analysis. Therefore, the current study did not limit the size of the lesions. Fifth, The study’s lack of pathological correlation was a limitation. Since this was a retrospective study, we could not assess the pathological features of CHAs. This could be an intriguing future direction.

## Conclusion

Finally, CHA imaging characteristics may increase CT diagnostic performance even further in the differential diagnosis of RCC and RO. When a renal mass with CHA is found, a longer delay scanning duration should be used to assess the enhancement inversion of CHA. This is particularly important if RO is suspected.

## Data Availability

The datasets used and/or analysed during the current study are available from the corresponding author on reasonable request.

## Abbreviations

RO: Renal oncocytoma
RCC: Renal cell carcinoma
ccRCC: Clear cell renal cell carcinoma
chRCC: Chromophobe renal cell carcinoma
pRCC: Papillary renal cell carcinoma
CHA: Central hypodense area
PTP: Peripheral tumor parenchyma
CMP: Corticomedullary phase
NP: Nephrographic phase
EP: Excretory phase
LSR: Long-to-short-axis ratio
AVT: Attenuation value of tumor
AVC: Attenuation value of the cortex
RER: Relative enhancement ratio
ROI: Regions of interest
PPV: Positive predictive value
AUC: Area under the curve
